# Essential and toxic elements intake from botanical extracts: a probabilistic risk–benefit evaluation within the Italian dietary context

**DOI:** 10.1002/jsfa.70494

**Published:** 2026-02-03

**Authors:** Giovanni Tommaso Lanza, Maria Olga Varrà, Lenka Husáková, Martina Piroutková, Jan Patočka, Emanuela Zanardi

**Affiliations:** ^1^ Department of Food and Drug University of Parma Parma Italy; ^2^ Department of Analytical Chemistry, Faculty of Chemical Technology University of Pardubice Pardubice Czech Republic

**Keywords:** food safety, essential elements, toxic metals, Monte Carlo simulation, risk–benefit assessment, cumulative risk

## Abstract

**BACKGROUND:**

Botanical extracts are widely consumed for their claimed health benefits, yet their safety profile with respect to chronic consumption remains poorly characterized. Understanding the potential health risks associated with their inorganic content is a crucial issue for ensuring safe use, along with a characterization of the concurrent nutritional contribution of the mineral component.

**RESULTS:**

The present study aimed to quantitatively assess exposure levels and potential health impacts of chronic intake of ten essential (Ca, K, P, Fe, Mg and Zn) and potentially toxic (Al, As, Ni and Pb) elements through the consumption of botanical extracts (*n* = 25) among Italian adults. A probabilistic approach was employed to estimate exposure levels and both risk and benefit metrics. Results indicated that botanicals alone contributed only minimally to mineral intakes, with 5^th^ to 95^th^ percentile (P5–P95) ranges covering 0.01–16.80% of the dietary reference values. Exposure to inorganic As (*i*As) raised health concerns because margin of exposure (MOE) values for skin cancer a ranged between 0.05 and 80.50 (P5–P95). When botanical extracts were considered alongside the baseline reference diet, Pb intake also raised concern because MOEs for nephrotoxic and cardiovascular effects fell below the critical threshold of 10. Similarly, cumulative exposure to Al, *i*As, Fe, Ni and Zn revealed potential non‐carcinogenic risks (mean hazard index > 1) only when considering the consumption of botanicals in addition to the baseline diet.

**CONCLUSION:**

The findings of the present study underscore the importance of the cautious dietary use of botanical extracts because of potential risks that may outweigh the presumed benefits. © 2026 The Author(s). *Journal of the Science of Food and Agriculture* published by John Wiley & Sons Ltd on behalf of Society of Chemical Industry.

## INTRODUCTION

Botanicals are plant materials that, when subjected to extraction, drying, pressing, fractionation, purification, concentration or fermentation processes, are referred to as ‘botanicals preparations’^1^ and represent concentrated sources of bioactive compounds used across the pharmaceutical, nutraceutical, cosmetic and food sectors.^2^, ^3^ In recent years, the consumption of botanicals has grown significantly, particularly in Western countries, driven by perceived health benefits, broad market availability as dietary supplements and food ingredients, and the misconception that natural products are inherently safe.^4^, ^5^ As a result, the simultaneous use of multiple botanicals is now common,^6^, ^7^ supported by claims of metabolic, cardiovascular, immune and anti‐inflammatory benefits.^8^

The concentration of essential and non‐essential (hereunder ‘potentially toxic’) elements in commercial botanical products varies according to several factors, including plant species, geographical origin, climate, soil composition, water quality and agricultural practices.^9^, ^10^ Post‐harvest handling can further alter element concentrations as a result of oxidation, degradation or contamination from equipment.^11^ In the European Union, several instances of contamination involving toxic metals and metalloids have been reported through the Rapid Alert System for Food and Feed,^12^ with many cases linked to plants sourced from heavily industrialized regions.^13^, ^14^

Previous studies have already investigated the inorganic composition of botanical extracts, emphasizing their potential nutritional benefits from essential nutrients.^15^, ^16^, ^17^ Conversely, many studies have reported toxic elements at levels that may pose health risks to consumers upon chronic exposure.^18^, ^19^, ^20^, ^21^, ^22^, ^23^, ^24^, ^25^, ^26^ These contrasting findings reveal a complex and often contradictory scenario, leaving the overall risk–benefit profile of such products unclear.

Risk–benefit assessment (RBA) is a systematic approach designed to weigh both the potential adverse effects (risks) and the beneficial health impacts (benefits) of consuming a food, one of its components, or a dietary intervention, with the aim of supporting policymakers in making informed decisions.^27^ The methodology for conducting an RBA is not entirely standardized and can vary by context,^28^, ^29^ whereas limitations in toxicological data (especially for emerging contaminants and unassessed health effects) continue to constrain its wider application.^27^

Given the widespread use of botanical extracts for their purported health benefits and considering the potential concurrent presence of toxic elements, there is the need for a systematic RBA to determine whether the associated risks outweigh the benefits, or vice versa, and to clarify their overall impact on consumers. Additionally, because simultaneous exposure to multiple toxic elements from botanicals is not well understood, the cumulative risk still remains uncertain.

The main objectives of the present study were to quantitively assess the dietary exposure of Italian adults to essential (Ca, K, P, Fe, Mg and Zn) and potentially toxic (Al, *i*As, Ni and Pb) elements resulting from the inclusion of botanical extracts in the diet. The study also aimed to assess both the possible health benefits and adverse effects of these exposures in comparison with a reference diet that does not include regular botanical intake. Additionally, a cumulative risk assessment was conducted to evaluate the combined non‐carcinogenic effects of multiple toxic elements. To achieve these objectives, a probabilistic Monte Carlo‐based RBA approach was used, allowing a more robust and realistic estimation of potential health outcomes.

## MATERIALS AND METHODS

### Sample collection

This study examined 25 commercial powdered botanical extracts, each from a different plant species and sold on the European market (Table 1). The declared country of origin for most samples was China, except for six products for which the origin was not clearly specified on the label (i.e. sea buckthorn extract, guarana extract, *Cordyceps sinensis* extract, skullcap extract and gardenia extract). Samples were deliberately selected to maximize taxonomic and compositional diversity, covering multiple plant families and representing botanical products commonly consumed in Europe. The inherent high degree of variability in their elemental composition was intentionally conceived and considered well‐suited for probabilistic analysis to capture a wide spectrum of real‐world exposure scenarios because the modeling approach adopted allowed incorporation and propagation of such variability.

**Table 1:** List of the plant species and commercial product name of the botanical extracts analyzed in the present study

Botanical species	Product name
*Gardenia jasminoides* Ellis	Gardenia extract
*Garcinia mangostana* L.	Garcinia Mangostana fruit rind extract
*Hippophae rhamnoides*	Sea Buckthorn extract
*Crategus monogyna*	Hawthorn extract
*Capsicum frutescens* L.	Cayenne extract
*Ligustrum lucidum* Ait.	Lingustrin lucidum extract
*Allium sativum* L.	Black garlic extract
*Orthosiphon aristatus*	Java tea extract
*Gymnema Sylvestre*	Gymnema Sylvestre extract (75%)
*Rosmarinus officinalis* L.	Rosemary extract
*Ginkgo biloba* L.	Ginkgo Biloba extract
*Camellia sinensis* L.	Green Tea Extract
*Taraxacum officinale*	Dandelion extract
*Juglans reggia*	Walnut extract
*Vitis vininfera* L.	Grape Skin extract
*Punica granatum* L.	Pomegranate extract
*Cordyceps sinensis*	Cordyceps sinensis extract (4:1)
*Ganoderma lucidum*	Reishi mushroom extract
*Cordyceps militaris*	Cordyceps extract (beta glucan)
*Beta vulgaris* L.	Beetroot powder E2.6
*Smilax china* L.	Sarsaparilla extract
*Scutellaria baicalensis*	Skullcap extract
*Urtica dioica* L.	Nettle Root extract
*Paullinia cupana* Kunth.	Guarana extract (10% caffeine)
*Monascus purpureus*	Red yeast rice extract

### Quantification of essential and potentially toxic elements

Ten elements, including essential nutrients (Ca, Fe, K, Mg, P and Zn) and toxicologically relevant metals and metalloids (Al, As, Ni and Pb), were considered. Selection of these elements was guided by three main criteria: (i) their potential to exert beneficial or adverse health effects; (ii) their consistently high yet variable concentrations across the analyzed samples; and (iii) the availability of literature data on their baseline dietary intake within the Italian population.

Element concentrations were quantified using inductively coupled plasma‐mass spectrometry, following a previously described method.^30^ Detailed analytical procedures are provided in the Supporting information (Section S1 and Tables S1 and S2).

### Risks and benefits evaluation: probabilistic modeling

RBA was conducted in accordance with the general principles and recommendations of the ‘Guidance on risk‐benefit assessment on foods’ of the European Food Safety Authority (EFSA).^27^ However, rather than evaluating the full chemical profile or the entire inorganic fraction of the botanicals, the assessment focused on specific nutritionally or toxicologically relevant elements for which analytical data were available. In line with EFSA guidance,^27^ the assessment corresponds to a Tier 2 RBA because it does not use common or composite risk–benefit metrics, although it does account for uncertainty and variability through probabilistic modeling.

To evaluate exposure levels of Italian adults to both essential nutrients and potentially toxic elements three distinct scenarios were formulated: (i) baseline dietary intake from the total diet excluding botanicals; (ii) chronic consumption of botanical extracts alone; and (iii) total exposure, reflecting the combined intake of toxic and essential elements from botanical extracts and the total diet.

As a probabilistic approach was adopted, input parameters were modeled as probability distributions or retrieved as mean values with their standard deviations (SDs), assuming specific distribution shapes based on data characteristics. The main input parameters included dietary consumption patterns (retrieved as mean and SD values, with assumed distribution shapes) and the measured elemental concentrations (modeled as probability distributions). Probabilistic simulations combined all input parameters and were conducted using the Monte Carlo method with 100 000 iterations. This approach generated full probabilistic intake, risk and benefit profiles, capturing both inter‐individual variability and parameter uncertainty, thereby providing a robust and realistic assessment than conventional deterministic single‐point estimates. Simulations were performed using the R ‘stats’ package and all data processing, statistical analyses, and visualizations were conducted in RStudio, version 2024.09.0 + 375, using R, version 4.3.2 (R Development Core Team, Vienna, Austria).

#### Formulation of a reference scenario for baseline dietary exposure

As the reference dietary scenario, the average daily intake of the studied elements among Italian adults was considered. This scenario served to characterize baseline dietary status and evaluate the impact of additional chronic exposure from botanical extracts.

Daily intakes of most elements were obtained from the 2012–2014 National Total Diet Study (TDS) carried out by the Italian National Institute of Health.^31^, ^32^ Specifically, data for *i*As^33^, Ni^34^, Ca, Fe, Zn, Al and Pb (Cubadda F, 2025, unpublished data) were retrieved as the mean ± SD and assumed to follow a lognormal distribution.

Daily intakes of K, Mg and P were available from the ‘Italian National Food Consumption Survey, INRAN‐SCAI 2005‐06’,^35^ retrieved and summarized as the weighted mean ± SD (i.e. to account for male and female proportions), and also assumed to follow a lognormal distribution, reflecting typical nutrient consumption patterns.^36^

Intake levels of all elements under the dietary reference scenario are provided in Table 2.

**Table 2:** Estimated daily intakes (EDIs) or weekly intakes (EWIs) of essential and potentially toxic elements (mean ± SD) in Italian adults

Element	EDI/EWI	Unit of measure	Reference
Essential			
K	3026 ± 875	mg day^−1^	Italian National Food Consumption Survey, INRAN‐SCAI 2005–06^35^
P	1269 ± 366	mg day^−1^	
Mg	279 ± 87	mg day^−1^	
Ca	914 ± 317	mg day^−1^	2012–2014 National Total Diet Study, Cubadda F. (2025), unpublished data
Fe	8.7 ± 2.9	mg day^−1^	
Zn	10.7 ± 3.3	mg day^−1^	
Potentially toxic			
iAs^†^	0.081 ± 0.030	mg kg BW^−1^ day^−1^	2012–2014 National Total Diet Study^33^
Ni	1.55 ± 0.72	mg kg BW^−1^ day^−1^	2012–2014 National Total Diet Study^34^
Al	58.2 ± 25.0	mg kg BW^−1^ day^−1^	2012–2014 National Total Diet Study, Cubadda F. (2025), unpublished data
Pb	0.160 ± 0.081	mg kg BW^−1^ day^−1^	

Note: *i*As: inorganic arsenic.

#### Retrieval of dietary consumption data of botanicals

Mean ± SD values for acute and chronic daily consumption of ‘herbal formulations and plant extracts’ by Italian adults (18–64 years, consumers only) were obtained from the EFSA Comprehensive European Food Consumption Database,^37^ which incorporates data from the latest Italian national dietary survey IV SCAI ADULT 2018–2020.^38^ Data were extracted as overall consumption rates (8.1 ± 19.4 g day^−1^, chronic) and as body weight–adjusted consumption rates [0.17 ± 0.35 g kg body weight (BW)^−1^ day^−1^, acute; 0.11 ± 0.27 g kg BW^−1^ day^−1^, chronic) and used to formulate the exposure scenario from botanical consumption.

Because consumption data were available only as summary statistics, they were assumed to follow a lognormal distribution.^36^, ^39^

#### Distribution fitting of elemental concentrations in botanical extracts

Element concentrations, determined experimentally, were fitted to statistical distributions using the Akaike information criterion (AIC) to identify the best‐fitting models. The delta AIC (ΔAIC) value was used to compare candidate distributions (normal, lognormal, exponential, Weibull and Pareto). According to established interpretation criteria, ΔAIC < 3 indicates minimal evidence favoring one model over another, values between 3 and 10 suggest moderate support, and ΔAIC < 10 provides evidence for the best‐fitting model. All calculations were performed in RStudio using the ‘fitdistrplus’ package.^40^

#### Retrieval of dietary reference values and health‐based guidance values

For Ca, K, Mg, Fe and Zn, benefit characterization was based on the comparison of dietary intakes with the following dietary reference values (DRVs) for adults, including adequate intake (AI), average requirement (AR), population reference intake (PRI) and upper intake level (UL). These values were obtained using the EFSA DRV Tool^41^ and relevant EFSA scientific opinions on each element.^42^, ^43^, ^44^, ^45^, ^46^, ^47^ For nutrients with sex‐specific DRVs (Mg, Fe and Zn), an average value was calculated and employed. For Zn, PRI values were available for four phytate intake levels (300, 600, 900 and 1200 mg day^−1^) and the arithmetic mean across these levels was used.

For Al, As, Ni and Pb, risk characterization was based on comparison with health‐based guidance values (HBGVs) and reference points (RPs), including tolerable daily intakes (TDIs), tolerable weekly intakes (TWIs), benchmark dose lower confidence limits (BMDLs) or the lowest‐observed‐adverse‐effect‐level (LOAEL). These were obtained from the EFSA Chemical Hazards Database (OpenFoodTox)^48^ and from the EFSA scientific opinions on each element.^49^, ^50^, ^51^, ^52^

A summary of the benefit and risk metrics applied in this study is provided in Table 3.

**Table 3:** Dietary reference values (DRVs), health‐based guidance values (HBGVs) and reference points (RPs) used for the benefit and risk characterization of essential and potentially toxic elements

Element	Benefit or risk metric	Value	Unit of measure	EFSA assessment: year^Ref^.
Essential				
K	AI^‡^	3500	mg day^−1^	2016^47^
P	AI^‡^	550	mg day^−1^	2015^46^
	UL^§^	3000	mg day^−1^	
Ca	PRI^†^	950	mg day^−1^	2015^43^
	UL	2500	mg day^−1^	
Mg	AI^‡^	325	mg day^−1^	2015^45^
	UL^§^	250	mg day^−1^	
Fe	PRI^†^	11	mg day^−1^	2015^44^
Zn	PRI^†^	11.5	mg day^−1^	2014^42^
	UL^§^	25	mg day^−1^	
Potentially toxic				
Al	TWI^ɠ^	1	mg kg BW^−1^ week^−1^	2008^49^
Ni	TDI^¶^	13	mg kg BW^−1^ day^−1^	2020^51^
	LOAEL^ᴪ^	4.3	mg kg BW^−1^ day^−1^	
*i*As^φ^	BMDL_05_^ɸ^ (skin cancer)	0.06	mg kg BW^−1^ day^−1^	2024^52^
Pb	BMDL_01_^ɸ^ (cardiovascular effects)	1.5	mg kg BW^−1^ day^−1^	2010^50^
	BMDL_10_^ɸ^ (nephrotoxicity)	0.63	mg kg BW^−1^ day^−1^	

*Note*: ^‡^AI, adequate intake; ^†^PRI, population reference intake; ^§^UL, upper intake level; ^ɠ^TWI, tolerable weekly intake; ^¶^TDI, tolerable daily intake; ^ᴪ^LOAEL, lowest‐observed‐adverse‐effect‐level; ^φ^ *i*As, inorganic arsenic; ^ɸ^BMDL, benchmark dose lower confidence limit.

#### Exposure assessment and risk/benefit characterization

The estimated daily intakes (EDIs) of Ca, K, Mg, Fe and Zn (mg day^−1^) from chronic consumption of botanicals were calculated using input Eqn (1) subjected to Monte Carlo simulations:

(1) $$\text{EDIs}\left(\mathrm{mg}\;{\mathrm{day}}^{-1}\right)=\;\frac{\text{Element concentration}\left(\mathrm{mg}\;{\mathrm{kg}}^{-1}\right)\;\times \;\text{Food consumption rate}\left({\text{g day}}^{-1}\right)}{1000}$$

To characterize the associated beneficial effects of nutrient intakes under the three formulated scenarios, the probability distributions of EDIs were compared with the DRVs for each nutrient. The percentage contribution to the DRVs (% AI or % PRI) were calculated using Eqns (2) and (3), both subjected to Monte Carlo simulations:

(2) $$\%\mathrm{DRV}\;\frac{{\mathrm{EDI}}_{a}\;\left(\text{or},\;,{\mathrm{EDI}}_{b}\right)}{\mathrm{DRV}}\;\times \;100$$

(3) $$\%{\mathrm{DRV}}_{\mathrm{tot}}\;=\;\frac{{\mathrm{EDI}}_{a}\;+\;{\mathrm{EDI}}_{b}}{\mathrm{DRV}}\;\times \;100$$

where EDI_a_ is the exposure deriving from the sole consumption of botanical extracts and EDI_b_ is the exposure deriving from the sole baseline reference scenario.

The EDIs of As, Ni, Al and Pb (μg kg BW^−1^ day^−1^) from chronic consumption of botanicals were calculated using input Eqn (4). The estimated weekly intake (EWI) of Al (μg kg BW^−1^ week^−1^) was then derived using Eqn (5) to allow comparison with its HBGV value (expressed on a weekly basis). EDIs of As, Ni and Pb and the EWI of Al were all subjected to Monte Carlo simulations.

(4) $$\mathrm{EDI}\left(\mathrm{\mu g}\;\mathrm{kg}\;{\mathrm{BW}}^{-1}\;{\mathrm{day}}^{-1}\right)=\begin{array}{l} \frac{\text{Element concentration}\left(\mathrm{\mu g}\;{\mathrm{kg}}^{-1}\right)}{1000}\\ \times \;\text{food consumption rate}\left(g\;\mathrm{da}y^{-1}\right) \end{array}$$

(5) $${\mathrm{EWI}}_{\mathrm{Al}}\;=\;{\mathrm{EDI}}_{\mathrm{Al}}\;\times \;7$$

To characterize the risk associated with chronic toxic element intake under the three formulated scenarios, the probability distributions of EDIs or EWIs were compared with the HBGVs or RPs established for these elements. Percentage contributions to the TDI (% TDI, for Ni) or TWI (% TWI, for Al) and the margin of exposure (MOE, for *i*As and Pb), were calculated using Eqns (6) and (7), both subjected to Monte Carlo simulations:

(6) $$\%\mathrm{TDI}\;\text{or}\%\mathrm{TWI}\;=\;\frac{\mathrm{EDI}\;\text{or}\;\mathrm{EWI}}{\text{HBGV}}\;\times \;100$$

(7) $$\mathrm{MOE}\;=\;\frac{\text{BMDL}}{\mathrm{EDI}}$$

Because oral exposure to Ni may also induce acute adverse effects in sensitized individuals, acute exposure and risk characterization were also addressed using Eqns (8) and (9), respectively (both subjected to Monte Carlo simulations):

(8) $$\text{Acute exposure}\left(\mathrm{\mu g}\;\mathrm{kg}\;bw^{-1}\right)\;=\;\frac{\mathrm{Ni}\;\text{concentration}\left(\mathrm{\mu g}\;kg^{-1}\right)\;\times \;\text{Acute food consumption}\;g\;\mathrm{kg}\;bw^{-1}}{1000}$$

(9) $$\mathrm{MOE}\;=\;\frac{\text{LOAEL}}{\text{Acute exposure}}$$

where the lowest‐observed‐adverse‐effect level (LOAEL) equal to 4.3 μg kg BW^−1^ (associated with systemic contact dermatitis) was employed as the RP and an MOE < 30 was considered indicative of potential health concerns.^51^ To determine which input parameters mostly influenced exposure, risk, or benefit outcomes, a sensitivity analysis was performed. This analysis focused only on estimates from chronic consumption of botanical extracts because the study aimed to assess botanicals specifically rather than total dietary intake. Spearman rank correlation coefficients (*ρ*), ranging from −1 to +1, were used to evaluate the relative influence of each modeled input variable, with higher absolute *ρ* values indicating greater contributions to variability in predicted exposure and identifying the main determinants of risks or benefits.

### Cumulative risk assessment

The non‐carcinogenic cumulative risk from chronic intake of Al, As, Ni, Fe and Zn was estimated by calculating the target hazard quotient (THQ) of each element and the hazard index (HI) as the sum of individual THQs. For this purpose, the following reference doses (RfDs), as established by the United States Environmental Protection Agency (US EPA), were used: 1.00 mg kg BW^−1^ day^−1^ for Al; 0.0003 mg kg BW^−1^ day^−1^ for *i*As; 0.7 mg kg BW^−1^ day^−1^ for Fe; 0.02 mg kg BW^−1^ day^−1^ for Ni; and 0.30 mg kg BW^−1^ day^−1^ for Zn.^53^ THQs were calculated using Eqns (10) and (11), both subjected to Monte Carlo simulations:

(10) $$\mathrm{THQ}\;=\;\frac{\mathrm{EDI}}{\mathrm{RfD}}\;\times \;\text{Exposure factor}$$

(11) $$\text{Exposure factor}\;=\;\frac{\mathrm{Ef}\;\times \;\mathrm{Ed}}{\mathrm{At}}$$

where *Ef* is the exposure frequency to botanicals in days per year (modeled as a truncated normal distribution; mean = 182.5 days; SD = 70 days; range = 1–365 days), *Ed* is the exposure duration in years (modeled as a truncated normal distribution; mean = 23 years; SD = 10 years; range = 1–46 years) and *At* is the averaging time (assumed to be equal to *Ed* × 365 days), as supported by the US EPA and ATSDR guidance for non‐carcinogenic cumulative risk assessment.^54^, ^55^

Dose addition was used as the default model, following US EPA guidance for health risk assessment of chemical mixtures (including metals) when there is no clear evidence suggesting response addition is more appropriate.^55^ Consequently, the HI parameter was calculated by the sum of all the individual THQ values.

## RESULTS AND DISCUSSION

### Elemental concentrations and distributions in botanical extracts

Elemental concentrations measured in the botanical extracts are presented in Table 4. The data exhibited substantial variability, with median values significantly lower than the means, indicating positively skewed distributions with long right tails. Major elements (K, P, Mg and Ca) spanned on average within the 1000–20 000 mg kg^−1^ range, whereas trace elements (Fe, Zn) were within 10–50 mg kg^−1^ (Table 4). Among potentially toxic elements, As was the most abundant, with a median concentration of 649 μg kg^−1^ (Table 4).

**Table 4:** Concentrations of essential (K, P, Ca, Mg, Fe and Zn) and potentially toxic (Al, Ni, As and Pb) elements quantified across botanical extracts (*n* = 25)

Element	Unit of measure	Mean ± SD	Median	5th to 95th percentile (P5–P95)
Essential				
K	mg kg^−1^	16 876 ± 31 000	8337	398–58 406
P	mg kg^−1^	1605 ± 2000	986	76–5009
Ca	mg kg^−1^	1400 ± 2300	756	42–4857
Mg	mg kg^−1^	878 ± 1300	506	35–2820
Fe	mg kg^−1^	55 ± 55	38	0–165
Zn	mg kg^−1^	9.0 ± 9.0	6.2	0.0–26.9
Potentially toxic				
Al	mg kg^−1^	28 ± 28	20	0–85
Ni	μg kg^−1^	2433 ± 7400	719	13–9478
As	μg kg^−1^	3026 ± 18 000	649	7–11 623
Pb	μg kg^−1^	128 ± 220	66	3–438

The results of the AIC test used to identify the best‐fitting distributions for elemental concentrations are provided in the Supporting information (Table S3). As, Ca, K, Mg, Ni, P and Pb concentrations were best described by lognormal distributions, whereas Fe, N, and Al concentrations comprised exponential distributions. These fitted models were subsequently used in the probabilistic exposure assessments.

The observed variability in essential and toxic element concentrations reflects the heterogeneous composition of the 25 botanical extracts analyzed, which encompassed different plant species and parts (leaves, fruits, peels and seeds) from multiple producers or manufacturers.^10^, ^30^, ^56^ Rather than being a limitation, this variability should be considered a strength because it enabled the simulation of a broad range of probabilistic exposure scenarios.

Currently, Europe has not established maximum levels (MLs) for potentially toxic elements in botanical extracts themselves^57^. Where available, MLs set for the source raw materials or final food supplement products should be used as reference benchmarks. For Pb, a MLs of 3.0 mg kg^−1^ applies to food supplements^57^ and none of the analyzed samples exceeded this threshold. For *i*As, existing MLs in plant‐based products range from 0.020 mg kg^−1^ (some fruit juices) to 0.30 mg kg^−1^ (certain rice products).^57^ Notably, dandelion and walnut extracts were identified as the most contaminated, with mean As concentrations far above these limits (2.2 and 69 mg kg^−1^, respectively). MLs for Ni have been recently established in specific food, with limits in products relevant for this study ranging from 0.25–0.40 mg kg^−1^ (fruit juices, fruiting and stem vegetables) to 10–15 10 mg kg^−1^ (nuts, dry beans, soybeans).^57^ No MLs currently exist at the European level for Al in food products.^57^ However, the high concentrations observed, the frequent Al occurrence in plant‐based products,^21^, ^58^, ^59^ and the potential health risks from chronic exposure, underscore the need to consider these elements for inclusion in future regulatory frameworks.

### Exposure assessment to essential elements and benefit characterization

Under the reference dietary scenario (Table 2), baseline EDIs of P (1269 ± 366 mg day^−1^) contributed 231 ± 67% of the AI of this element, although remaining below the tolerable upper intake level of 3000 mg day^−1^. This may indicate a high likelihood of adequate P intake across the adult population. P is essential for many physiological processes, including bone mineralization, energy metabolism and cellular function.^46^ The EDIs of the other essential nutrients were slightly below their respective DRVs but characterized by a high degree of variability (Table 2). Because nutrient intake distributions were modeled as lognormal, most of the population is expected to have intakes below the mean as a result of the right‐skewed nature of the distribution.^36^ This pattern suggests that, except for P, a substantial proportion of individuals may be at risk of inadequate intake of all essential elements from the baseline diet alone. Fe and Zn are essential for oxygen transport, energy production, immune function and enzymatic activity, with deficiencies potentially causing anemia and impaired immune response.^42^, ^44^ Chronic inadequate Mg intake may lead to neurological or cardiac issues, whereas K intakes below the AI are associated with increased risk of stroke.^45^, ^47^ Prolonged insufficient Ca intake may reduce bone mineral density, increasing the risk of osteoporosis and bone fractures.^43^

The EDIs of essential elements from the exclusive consumption of botanical extracts and from the scenario combining botanicals consumption plus baseline diet are reported in Table 5, whereas the percentage contributions to DRVs deriving from sole consumption of botanicals are shown in Table 6. As observed, nutrient intakes from the sole consumption of botanicals provided a minimal contribution to DRVs, with median values generally below the 1% and 95^th^ percentile (P95) values reaching a maximum of 17% (Table 6). Therefore, botanical extracts contribute only modestly to recommended nutrient intakes and, although they may help address minor nutritional shortfalls (especially in individuals with marginal deficiencies),^60^, ^61^ this effect is incidental because it is not the primary intended function of these products.

**Table 5:** Estimated daily intakes (EDIs) or weekly intakes (EWIs) [mean ± SD, median and 5^th^ to 95^th^ percentile (P5–P95) values] of essential and potentially toxic elements in Italian adults through the sole chronic consumption of botanical extracts and though the whole diet supplemented with botanical extracts (for Ni, both the chronic and the acute^§^ EDI were assessed)

Element	Unit of measure	EDI/EWI (botanicals only)			Total EDI/EWI (reference scenario + botanicals)			Average contribution (%) of botanicals to total EDI/EWI of the reference scenario + botanicals
		Mean ± SD	Median	P5–P95	Mean ± SD	Median	P5–P95	Mean ± SD
Essential								
Ca	mg day^−1^	11 ± 45	2	0–44	926 ± 321	876	504–1522	1.2 ± 3.4
K	mg day^−1^	135 ± 608	26	1–513	3160 ± 1069	3016	1891–4895	3.4 ± 7.5
P	mg day^−1^	13 ± 49	3	0–51	1283 ± 370	1231	775–1962	1.0 ± 2.7
Mg	mg day^−1^	7 ± 27	2	0–27	286 ± 91	273	166–449	2.2 ± 5.2
Fe	mg day^−1^	0.4 ± 1.5	0.1	0.0–1.8	9.1 ± 3.2	8.6	5.1–14.7	4.2 ± 8.0
Zn	mg day^−1^	0.07 ± 0.28	0.02	0.01–0.29	10.8 ± 3.3	10.29	6.3–16.8	0.7 ± 1.9
Potentially toxic								
Al	mg kg BW^−1^ week^−1^	0.024 ± 0.092	0.006	0.000–0.091	0.43 ± 0.19	0.39	0.20–0.77	4.7 ± 8.8
Ni	μg kg BW^−1^ day^−1^	0.3 ± 2.2	0.0	0.0–0.1	1.8 ± 2.0	1.6	0.1–3.5	9 ± 15
Ni (acute)^§^	μg kg BW^−1^	0.4 ± 2.8	0.1	0.0 ± 1.5	/	/	/	/
*i*As ^†^	mg kg BW^−1^ day^−1^	0.4 ± 3.2	0.0	0.0–1.1	0.4 ± 3.3	0.12	0.0–1.2	37 ± 32
Pb	mg kg BW^−1^ day^−1^	0.023 ± 0.082	0.003	0.000–0.056	0.18 ± 0.11	0.15	0.07–0.34	7 ± 12

*Note*: ^§^Acute exposure to Ni: calculated only for the scenario considering the sole consumption of botanicals; ^†^ *i*As: inorganic arsenic.

**Table 6:** Percentage contributions [mean ± SD, median and 5^th^–95^th^ percentile (P5–P95) values] of the of chronic consumption of botanical extracts by Italian adults to Ca, K, Mg, P, Fe and Zn benefit metrics and to Al and Ni risk metrics [margin of exposure (MOE) values are provided for the Ni (under the acute exposure scenario), inorganic As (*i*As) and Pb (both under chronic exposure scenarios)]

Element	Mean ± SD	Median	P5–P95	Health metric
Essential				
Ca	1.2 ± 4.9	0.3	0.0–4.7	%PRI
K	4 ± 20	1.0	0–15	%AI
Mg	2.2 ± 8.6	0.5	0.0–8.8	%AI
P	2.4 ± 8.6	0.6	0.0–9.3	%AI
Fe	4 ± 13	1.0	0–17	%PRI
Zn	0.6 ± 2.0	0.2	0.0–2.6	%PRI
Potentially toxic				
Al	2.3 ± 8.2	0.5	0.0–9.3	%TWI
Ni	2 ± 15	0.2	0–7	%TDI
Ni (acute scenario) ^ɸ^	637 ± 7766	82	3–2231	MOE
*i*As ^†^	25 ± 207	2	0–81	MOE
Pb^§^	1087 ± 4625	218	12–4225	MOE
Pb^¶^	2588 ± 11 012	519	27–10 059	MOE

*Note*: ^ɸ^MOE calculated using the LOAEL for eczematous flare‐up skin reactions in nickel‐sensitized individuals; ^†^MOE calculated using the BMDL_05_ for skin cancer; ^§^MOE calculated using the BMDL_10_ for nephrotoxicity; ^¶^MOE calculated using the BMDL_01_ for cardiovascular diseases.

Botanical extracts, infusions and various herbal formulations are recognized sources of essential minerals and have been linked to a range of health benefits.^62^, ^63^, ^64^, ^65^, ^66^ However, few studies have quantitatively estimated the chronic intake of such nutrients from these products. For example, Muhammad *et al*.^67^ reported lower EDIs of K and Ca but higher EDIs of Fe (3.7 mg day^−1^) and Zn (7.5 mg day^−1^) from *Viola betonicifolia* compared to the botanicals analyzed in the present study. Ayurvedic extracts were also reported to be safe regarding heavy metals at the same time as providing adequate essential minerals, particularly Fe (EDIs up to 2.7 mg day^−1^).^24^ Conversely, other herbal extracts such as *Urtica dioica* (also analyzed in the present study), were reported to provide negligible amounts of Ca, Mg, Fe, Zn and Cu for human nutrition.^68^ Importantly, the bioavailability of essential elements from plant materials can vary widely. Therefore, high concentrations do not necessarily translate into high absorption in humans. Indeed, bioaccessibility and bioavailability depend on the chemical form of the element, as well as on interactions with other plant constituents (including phytates, oxalates and polyphenols), which can reduce mineral absorption.^69^ From another perspective, despite limited bioavailability, essential elements can still help reduce the absorption and accumulation of toxic metals such as Pb and Cd, primarily through competitive interactions among divalent metals for the same intestinal transporters.^70^, ^71^

**Figure 1:**
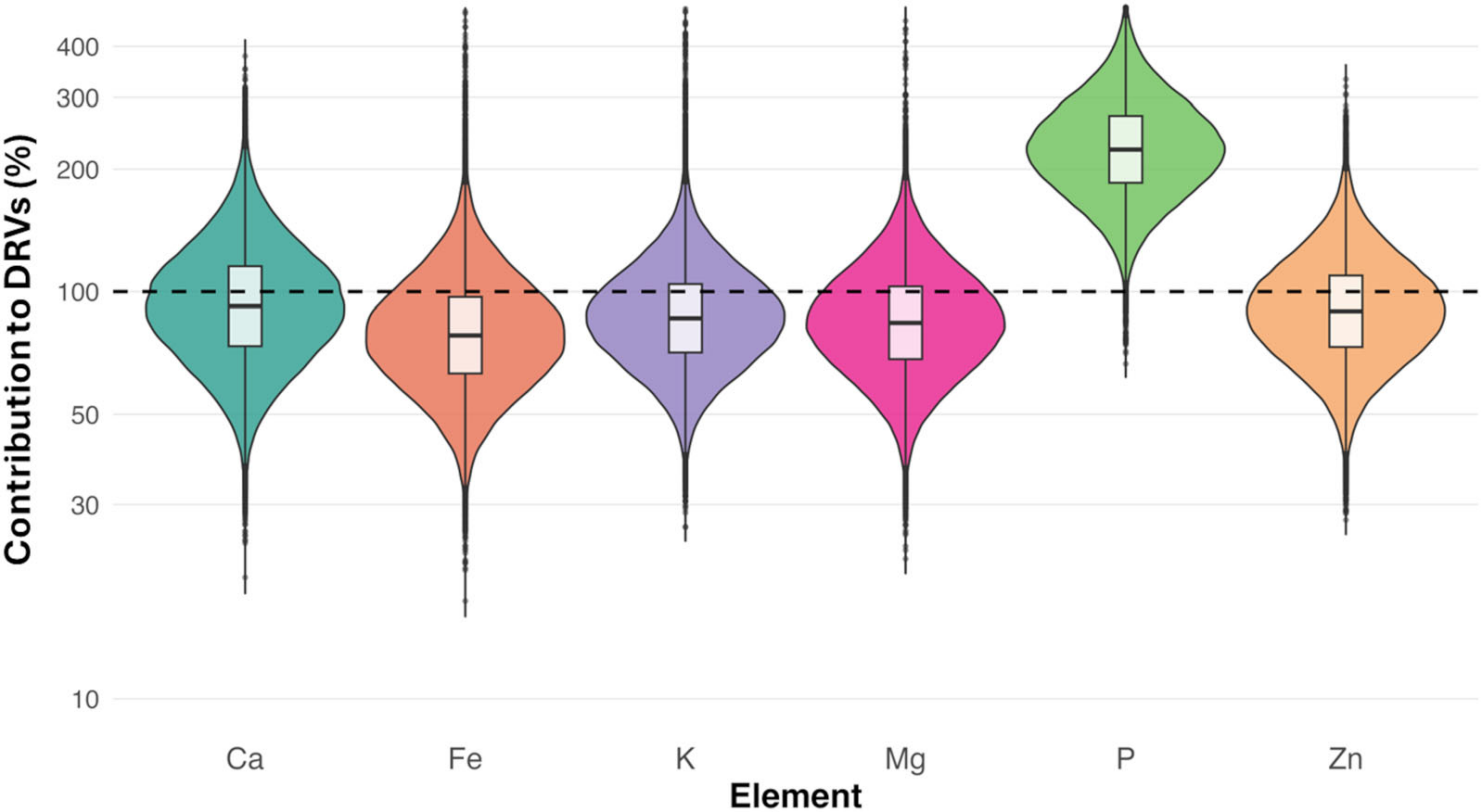
Distribution of the calculated percentage contribution (%) of dietary intakes of essential nutrients to their respective dietary reference values (DRVs), resulting from the combined consumption of botanical extracts and the total (reference) diet.

Fig. 1 shows the probability distributions of the total percentage contribution to the DRVs of Ca, K, Mg, P, Fe and Zn when combining intakes from the reference diet and botanical extracts. P intakes consistently exceeded nutritional requirements across the population, with most values between 150% and 250% of the DRV. This may suggest that, even without botanical supplementation, P intake from the diet is generally sufficient. In contrast, Ca, K, Mg and Zn to DRVs median intake contributed less than 100% of the DRVs, suggesting that a significant portion may be at risk of suboptimal intake.

Overall, these results underscore that, although botanical extracts may provide beneficial minerals, they are insufficient on their own to fully address potential dietary inadequacies.

### Exposure assessment to potentially toxic elements and risk characterization

The EDIs of As, Ni and Pb and EWIs of Al from the baseline reference scenario are presented in Table 2. The highest value was observed for Al (58.2 ± 25.0 μg kg BW^−1^ day^−1^), whereas the lowest was observed for *i*As (0.081 ± 0.030 μg kg BW^−1^ day^−1^) (Table 2). These chronic intake levels contributed less than 100% of the TWI of Al (41%) and TDI of Ni (12%), indicating that adults are unlikely to face health risks such neurotoxicity, embryotoxicity, reproductive toxicity, or immunotoxicity from the baseline diet.^49^, ^51^

Chronic consumption of botanical extracts alone contributed less than 1% to the TWI of Al and the TDI of Ni at the median intake level, remaining below 10% even at the P95 of the entire simulated distribution values (Table 6). Therefore, the dietary consumption of botanicals appears safe regarding Al and Ni toxicity, although this contrasts with some findings reported in earlier studies.^14^, ^21^, ^64^

**Figure 2:**
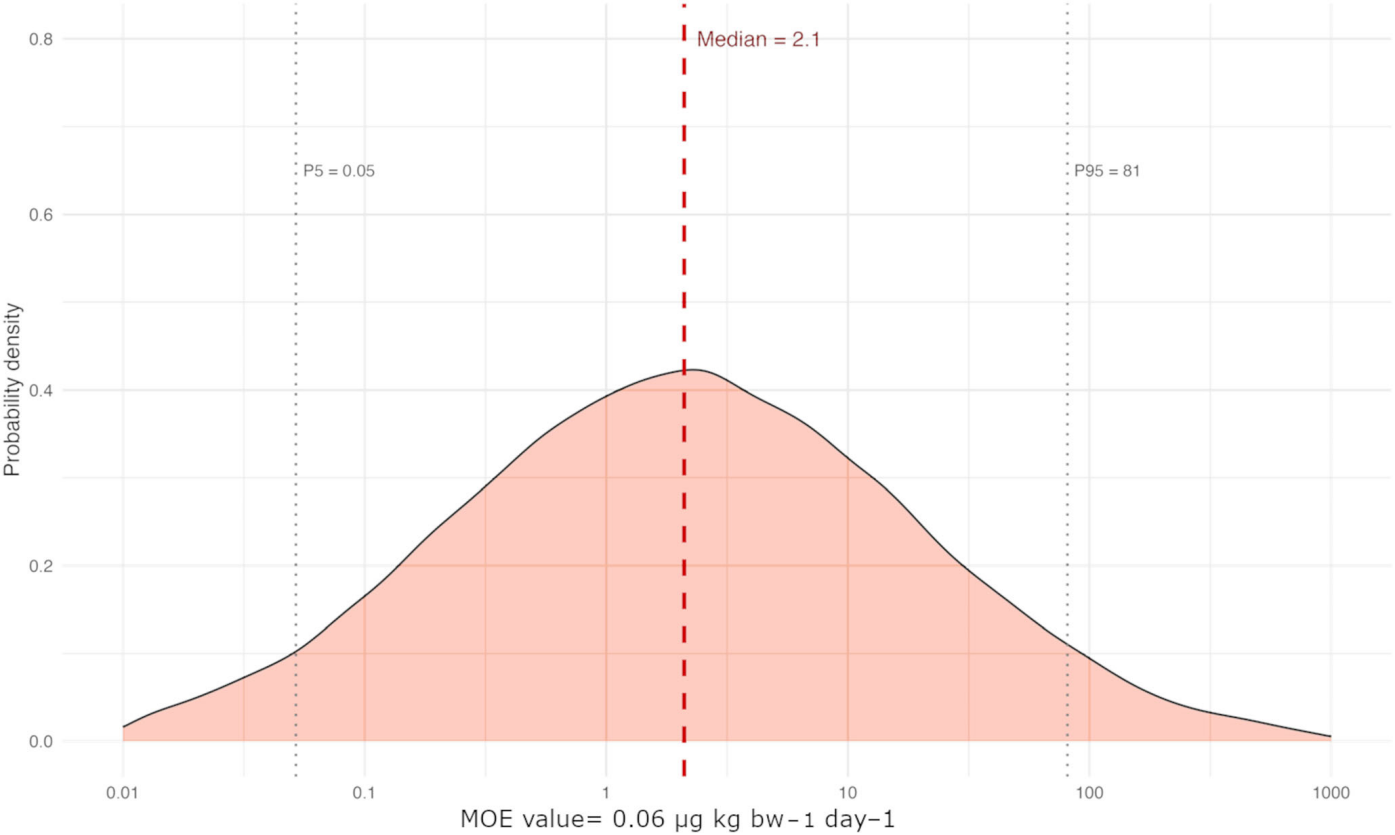
Probability distribution of margin of exposures (MOEs, logarithmic scale) to inorganic arsenic resulting from the consumption of botanical extracts and associated with a 5% relative increase of skin cancer incidence.

Tables 5 and 6 present intake levels and associated risk estimates from the exclusive consumption of botanicals. Among the evaluated elements, *i*As emerged as the primary concern. As shown in Table 6 and further illustrated in Fig. 2 (displaying the entire probability distribution curve of simulated MOEs on a logarithmic scale), median MOEs were below 10, and fell below 1 when considering the P5 values of the simulations. These results align with previous studies that identify *i*As as a major contaminant in botanicals,^72^, ^73^, ^74^ potentially exposing 54% of adults to carcinogenic risk.^26^, ^75^

Sensitivity analysis revealed that the MOE to *i*As had moderate‐to‐strong negative correlations with both the consumption rate of botanicals (*ρ* = −0.59) and *i*As concentrations in the samples (*ρ* = −0.77), consistent with the expectation that higher exposure results in lower MOEs. In this context, although *i*As was not directly measured in the samples, it is important to note that a large proportion of total As in botanicals is typically inorganic.^74^ Therefore, these results indicate a potentially serious health risk and underscore the critical need to minimize exposure to this contaminant as much as reasonably achievable.^52^

Regarding exposure to Pb, MOEs calculated under the reference scenario were substantially below 10 for both nephrotoxicity (95% of MOEs < 10) and cardiovascular toxicity (46% of MOEs < 10) endpoints, indicating that baseline dietary intake alone may pose health risks.^50^, ^76^ By contrast, exposure from botanicals alone did not raise concerns because all simulated MOEs exceeded the safety threshold of 10 (Table 6). However, when combined with background dietary intake, Pb exposure from botanicals resulted in median MOEs of 4.1 (nephrotoxicity) and 9.7 (cardiovascular effects), with even lower values of the P5 estimates (1.8 and 4.4, respectively). Hence, these results indicate that the already problematic baseline Pb exposure can be further worsened by botanical consumption, emphasizing the importance of limiting total dietary Pb intake.^77^ This is consistent with previous studies identifying Pb as a common contaminant in plant‐derived materials.^19^, ^74^, ^78^, ^79^, ^80^

Although the present assessment focused on adults, the implications for vulnerable subgroups are likely more concerning. Indeed, younger individuals have higher exposure relative to body weight and less mature detoxification systems, which increase their sensitivity to the harmful effects of *i*As and Pb. Pregnant and breastfeeding women may also face heightened risks because even small increases in exposure can negatively affect fetal and early‐life development. Therefore, although risks are already evident for adults, the findings emphasize the importance of careful consideration for these more susceptible populations.

#### Acute exposure to Ni

Acute exposure to Ni from the consumption of botanical extracts showed substantial variability, with intake estimates up to 1.5 μg kg BW^−1^ (Table 5). These exposure levels led to MOEs spanning from 3 (P5) to 2231 (P95), with a median value of 82 (Table 6; see also Supporting information, Fig. S1). Notably, 32% of the simulated MOEs fell below the threshold of 30, indicating that a substantial portion of Ni‐sensitized individuals could experience acute adverse effects from the Ni content of botanicals (see Supporting information, Fig. S1). This finding is of particular concern given that approximately 15% of the European population (especially women) is affected by Ni sensitization^51^ and that a subset of these Ni‐sensitized individuals is affected by systemic contact dermatitis.

#### Cumulative dietary risk assessment

A probabilistic estimation of cumulative non‐carcinogenic risks from simultaneous exposure to Al, *i*As, Fe, Ni and Zn was further conducted using the HI as a composite risk metric, where values below 1 indicate low or unlikely risk.^54^, ^55^

The HI resulting from the consumption of botanicals alone ranged from 0.05 (median) to 1.95 (P95). The cumulative risk was mainly driven by the *i*As (THQ = 0.65), which contributed approximately 98% of the mean HI. Conversely, THQs of Al, Fe, Ni and Zn were all below 0.01, indicating minimal contribution to cumulative non‐carcinogenic risk.

**Figure 3:**
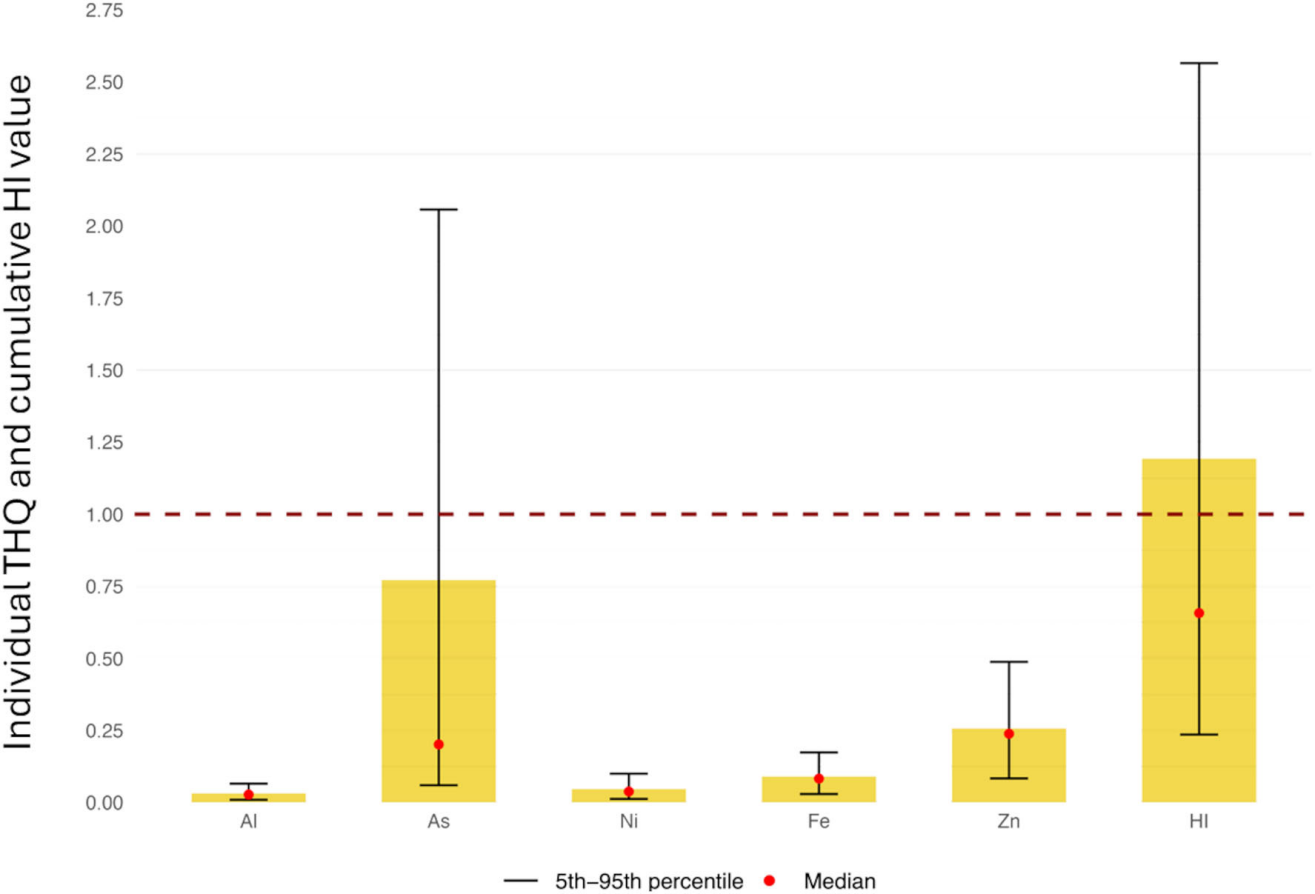
Target hazard quotient (THQ) values for individual toxic elements and hazard index (HI), representing the cumulative non‐carcinogenic risk from exposure to all assessed elements from the combined consumption of botanical extracts and the total (reference) diet.

When botanical consumption was combined with background dietary intake, the HI increased substantially, reaching a mean value higher than the threshold of 1 and P95 value reaching 2.6 (Fig. 3). Even under this combined scenario, *i*As remained the dominant contributor, accounting for 65% of the HI. Fe and Zn also contributed significantly, with P5–P95 THQ ranges of 0.03–0.17 and 0.08–0.49, respectively, whereas Ni and Al contributed less (average THQs below 0.04). Although high exposure to Zn and Fe is partially regulated by adaptive mechanisms such as reduced absorption and increased excretion, prolonged intakes that exceed homeostatic capacity can still lead to adverse and potentially irreversible biological effects^81^, including immunological alterations, impaired copper absorption and neurological issues.^42^, ^44^

Several studies have assessed the cumulative non‐carcinogenic risk from multiple elements in botanical and medicinal products. In some cases, HI values exceeded 1, with *i*As, Pb, Cd and Ni as main contributors.^19^, ^72^, ^74^, ^82^, ^83^ In other cases, plants from non‐industrialized regions or certain herbal products at medicinal doses previously showed HI values below 1, suggesting minimal risk from chronic exposure.^84^, ^85^

Overall, although exposure to individual elements may appear safe, combined chronic exposure can pose significant health risks. The substantial uncertainty observed in the results (particularly for *i*As) underscores many challenges in accurately assessing the risks more than the benefits associated with botanical consumption.

## CONCLUSIONS

The results of the present study reveal a complex interplay between modest nutritional benefits and non‐negligible toxicological risks from regular consumption of botanical extracts by Italian adults. Overall, botanicals contributed minimally to essential nutrient intake and their use should not be considered as a means to address potential nutritional deficiencies because they are neither intended nor marketed for this purpose. From a risk perspective, health concerns were evident from exposure to individual toxic elements (*i*As and Pb) and from cumulative exposure to Al, *i*As, Fe, Ni and Zn.

By providing a comprehensive assessment of both essential and toxic elements in botanicals, the present study aligns with several United Nations Sustainable Development Goals (SDGs) by informing nutritional adequacy (SDG 2), characterizing chronic exposure risks (SDG 3), and promoting responsible evaluation and management of chemical components in botanicals (SDG 12). However, some limitations should be acknowledged, including the exclusion of some toxic elements with similar endpoints and the exclusive focus on healthy adults, which may have led to an underestimation of the risks for more vulnerable populations. Therefore, future research should address these gaps and explore mitigation strategies to reduce contamination during production, processing, and formulation. From a policy perspective, there is a clear need for a harmonized and enforced regulatory framework for botanical extracts. Priorities should include establishing clear quality requirements, standardized composition guidelines and maximum allowable limits for multiple inorganic contaminants. Equally important is the development of public guidance to promote informed and safe use of these products, which should be effectively communicated through standardized and transparent labeling.

Together, these measures are crucial to ensure that the nutritional advantages of these botanical extracts are safely exploited without compromising consumer health.

## FUNDING

This work was supported by Univerzita Pardubice (Grant Number SGS_2025_001) and Università degli Studi di Parma, Project funded under the National Recovery and Resilience Plan (NRRP), Mission 4 Component 2 Investment 13 – Call for tender No 341 of 15/03/2022 of Ministero dell'Università e della Ricerca – NextGenerationEU Award Number: Project code PE0000003, Project title ‘Research and innovation network on food and nutrition Sustainability, Safety and Security – Working ON Foods’ (ONFoods).

This research was also undertaken within the framework of the ALIFAR project, supported by Ministero dell'Università e della Ricerca through the ‘Dipartimenti di Eccellenza 2023–2027’ program.

## CONFLICTS OF INTEREST

The authors declare that they have no conflicts of interest.

## Supporting information


**Table S1.** Accuracy and precision of ICP‐MS quantification of essential and potentially toxic elements in certified reference materials (CRMs).
**Table S2**. Calculated detection limits of the method (MLOD, μg kg^−1^) and quantification limits of the method (MLOQs, μg kg^−1^) of essential and potentially toxic elements with the use of Rh as internal standard.
**Table S3**. Results of the Akaike Information Criterion (AIC) test used to identify the best‐fitting probability distributions for concentration values of each element measured in botanical extracts.
**Figure S1**. Probability distribution of margin of exposure (MOE, logarithmic scale) values to nickel based on acute dietary consumption of botanical extracts associated with systemic contact dermatitis in nickel‐sensitized individuals.

## Data Availability

The data that support the findings of this study are available from the corresponding author upon reasonable request.
