# Supplementary material for: Essential and toxic elements intake from botanical extracts: a probabilistic risk–benefit evaluation within the Italian dietary context
Source: J Sci Food Agric. 2026 Feb 3;106(7):4060–72. doi: 10.1002/jsfa.70494 (PMC13067091; doi:10.1002/jsfa.70494)
Supplement: Supplementary file 1 — Table S1. Accuracy and precision of ICP‐MS quantification of essential and potentially toxic elements in certified reference materials (CRMs). Table S2. Calculated detection limits of the method (MLOD, μg kg−1) and quantification limits of the method (MLOQs, μg kg−1) of essential and potentially toxic elements with the use of Rh as internal standard. Table S3. Results of the Akaike Information Criterion (AIC) test used to identify the best‐fitting probability distributions for concentration values of each element measured in botanical extracts. Figure S1. Probability distribution of margin of exposure (MOE, logarithmic scale) values to nickel based on acute dietary consumption of botanical extracts associated with systemic contact dermatitis in nickel‐sensitized individuals. [file JSFA-106-4060-s001.docx]

***Supporting Information***

**Essential and toxic elements intake from botanical extracts: a probabilistic risk-benefit evaluation within the Italian dietary context**

Giovanni Tommaso LANZA ^a^, Maria Olga VARRà ^a *^, Lenka Husáková ^b^, Martina Piroutková ^b^, Jan Patočka ^b^, Emanuela ZANARDI ^a^

*^a^University of Parma, Department of Food and Drug, Strada del Taglio, 10, 43126 Parma, Italy*

*^b^University of Pardubice, Department of Analytical Chemistry, Faculty of Chemical Technology, Studentska 573 HB/D, Pardubice, CZ-532 10, Czech Republic*

***CORRESPONDING AUTHOR:**

E-mail address: [mariaolga.varra@unipr.it](mailto:mariaolga.varra@unipr.it) (M.O. Varrà)

**Supporting Section 1**

The quantification of Ca, Fe, K, Mg, P, Zn, Al, As, Ni, and Pb was performed using an Agilent 7900 instrument (Agilent Technologies, Santa Clara, CA, USA), following a previously developed and validated method.^31^ Briefly, powdered botanical samples were fully mineralized under controlled microwave-assisted conditions (Speedwave XPERT digestion unit, Berghof, Eningen, Germany) using a mixture of nitric acid (65% w/w, Lach-Ner, Neratovice, Czech Republic) diluted 1:3 (v/v) with ultra-pure water, and hydrogen peroxide (≥ 30% w/w; Fluka Chemie AG, Buchs, Switzerland). Essential elements (K, Mg, P, Ca, Fe, and Zn) were quantified in the 0–10 mg/L range, while potentially toxic elements (Al, As, Ni, and Pb) in the 0–100 µg L^-1^ range, using external calibration curves prepared from standard solutions (Analytika Ltd., Prague, Czech Republic; Supelco ICP multi-element standard solution IV, Merck, Darmstadt, Germany). A Rh solution (200 µg L^-1^, SCP Science, Montreal, Canada) was used as the internal standard to correct for instrumental drift and matrix effects. All calibration curves showed excellent linearity (coefficients of determination, R² > 0.999).

Quality assurance and quality control procedures included the analysis of three independent replicates per sample, as well as the inclusion of method blanks prepared using the same volumes and concentrations of digestion reagents, subjected to the same microwave digestion protocol, and diluted to the same final concentrations as the samples. Commercially available certified reference materials (CRMs) were also analyzed to assess trueness of quantification. These included: GBW 10052-Green Tea and GBW07603-Bush Leaves (National Institute of Metrology and Institute of Geophysical and Geochemical Exploration, Beijing, China); NCS ZC 73015-Milk Powder (National Research Centre for Certified Reference Materials, Beijing, China); CRM 12–2-04-Wheat Bread Flour and CRM 12–2–03 P-Lucerne (pb-anal, Kosice, Slovakia); BCR 679-White Cabbage (Community Bureau of Reference, Geel, Belgium). Intra-day and inter-day precision values were satisfactory for all elements (relative standard deviations, RSD% < 10%), and recovery values for all elements were mostly within 85–110% (Table S1, Supporting Information). Method sensitivity was assessed through method limits of detection (MLODs) and method limits of quantification (MLOQs), calculated as three and ten times the standard deviation of 10 method blank replicates, respectively, adjusted for the sample dilution factor. MLOD and MLOQ values obtained are reported in Table S2 (Supporting Information).

**Table S1.**

Accuracy and precision of ICP-MS quantification of essential and potentially toxic elements in certified reference materials (CRMs). Reported values include declared and measured values (mg kg^-1^, mean ± SD, n=3), recoveries (R%), and intra- and inter-day relative standard deviations (RSD%).

| **Analyte** | **CRM** | **Declared**  **value** | **Measured**  **value** | **R (%)** | **RSD (%)** | |
| --- | --- | --- | --- | --- | --- | --- |
|  |  |  |  |  | **Intra-day** | **Inter-day** |
| **Essential**  **elements** |  |  |  |  |  |  |
| Ca | CRM 12-2-04 Wheat bread flour | 292 ± 30 | 301 ± 22 | 103 | 3.60 | 3.37 |
|  | BCR 679 White Cabbage | 7768 ± 655 | 7415 ± 225 | 95 | 1.52 | 0.03 |
|  | NCS ZC 73015 Milk Powder | 9400 ± 300 | 9230 ± 229 | 98 | 1.24 | 1.47 |
|  | GBW 10052 Green Tea | 12100 ± 300 | 10544 ± 322 | 87 | 1.53 | 1.77 |
|  | GBW 07603 Bush Leaves | 16800 ± 1100 | 16823 ± 591 | 100 | 1.76 | 1.00 |
|  | CRM 12-2-03 Lucerne | 17500 ± 750 | 17540 ± 226 | 100 | 0.64 | 7.95 |
| Fe | NCS ZC 73015 Milk Powder | 7.8 ± 1.3 | 6.7 ± 0.6 | 85 | 4.36 | 4.55 |
|  | CRM 12-2-04 Wheat bread flour | 23.8 ± 1.5 | 23.50 ± 0.03 | 99 | 0.07 | 7.50 |
|  | BCR 679 White Cabbage | 55.0 ± 2.5 | 51.9 ± 4.2 | 94 | 4.04 | 0.99 |
|  | GBW 10052 Green Tea | 322 ± 23 | 286.0 ± 7.6 | 89 | 1.32 | 0.55 |
|  | CRM12-2-03 Lucerne | 355 ± 18 | 353 ± 25 | 99 | 3.58 | 5.22 |
|  | GBW 07603 Bush Leaves | 1070 ± 57 | 884 ±74 | 83 | 4.18 | 0.55 |
| K | CRM 12-2-04 Wheat bread flour | 2550 ± 100 | 2540 ± 145 | 99 | 2.85 | 3.82 |
|  | GBW 07603 Bush Leaves | 9200 ± 1000 | 8972 ± 138 | 98 | 0.77 | 5.67 |
|  | NCS ZC 73015 Milk Powder | 12500 ± 500 | 12530 ± 143 | 100 | 0.57 | 8.19 |
|  | GBW 10052 Green Tea | 15500 ± 700 | 14281 ± 819 | 92 | 2.87 | 1.22 |
|  | CRM 12-2-03 Lucerne | 18700 ± 650 | 18512 ± 781 | 99 | 2.11 | 0.23 |
| Mg | CRM 12-2-04 Wheat bread flour | 556 ± 29 | 548 ± 17 | 99 | 1.52 | 2.87 |
|  | NCS ZC 73015 Milk Powder | 960 ± 70 | 969 ± 77 | 101 | 3.97 | 4.38 |
|  | BCR 679 White Cabbage | 1362 ± 127 | 1198 ± 29 | 88 | 1.19 | 0.25 |
|  | GBW 10052 Green Tea | 2200 ± 80 | 1843 ± 38 | 84 | 1.04 | 2.12 |
|  | CRM12-2-03 Lucerne | 3520 ± 125 | 3507 ± 245 | 99 | 3.50 | 4.78 |
|  | GBW 07603 Bush Leaves | 4800 ± 400 | 4399 ± 120 | 92 | 1.36 | 1.65 |
| P | GBW 07603 Bush Leaves | 1000 ± 40 | 927 ± 42 | 93 | 2.28 | 1.90 |
|  | CRM 12-2-04 Wheat bread flour | 2280 ± 85 | 2167 ± 64 | 95 | 1.48 | 6.67 |
|  | GBW 10052 Green Tea | 2800 ± 100 | 2423 ± 95 | 87 | 1.95 | 1.32 |
|  | CRM 12-2-03 Lucerne | 3030 ± 90 | 3010 ± 214 | 99 | 3.55 | 7.00 |
|  | BCR 679 White Cabbage | 3307 ± 241 | 3126 ± 33 | 95 | 0.52 | 0.21 |
|  | NCS ZC 73015 Milk Powder | 7600 ± 300 | 7613 ± 214 | 100 | 1.40 | 3.16 |
| Zn | CRM 12-2-04 Wheat bread flour | 17.90 ± 0.75 | 17.10 ± 0.10 | 96 | 1.87 | 2.82 |
|  | NCS ZC 73015 Milk Powder | 34 ± 2 | 34 ± 5 | 99 | 6.91 | 5.66 |
|  | GBW 10052 Green Tea | 35 ± 2 | 34 ± 1 | 96 | 0.77 | 1.28 |
|  | GBW 07603 Bush Leaves | 55 ± 4 | 55 ± 1 | 99 | 0.72 | 0.24 |
|  | BCR 679 White Cabbage | 79.7 ± 2.7 | 77.1 ± 1.7 | 97 | 1.12 | 0.34 |
|  |  |  |  |  |  |  |
| **Analyte** | **CRM** | **Declared**  **value** | **Measured**  **value** | **R (%)** | **RSD (%)** | |
|  |  |  |  |  | **Intra-day** | **Inter-day** |
| **Toxic elements** |  |  |  |  |  |  |
| Al | CRM 12-2-04 Wheat bread flour | 3 | 3.36 ± 0.35 | 112 | 5.22 | 4.02 |
|  | CRM12-2-03 Lucerne | 330 | 335 ± 20 | 101 | 3.04 | 6.33 |
| As | BCR 679 White Cabbage | 6.7^c^ | 7.2 ± 0.7^c^ | 107 | 4.59 | 12.8 |
|  | CRM 12-2-04 Wheat bread flour | 0.0170 ± 0.0046 | 0.0177 ± 0.0004 | 104 | 1.09 | 8.23 |
|  | NCS ZC 73015 Milk Powder | 0.031 ± 0.007 | 0.031 ± 0.005 | 99 | 8.46 | 4.29 |
|  | CRM12-2-03 Lucerne | 0.262 ± 0.020 | 0.260 ± 0.018 | 99 | 3.48 | 2.75 |
|  | GBW 10052 Green Tea | 0.27 ± 0.05 | 0.263 ± 0.008 | 97 | 1.57 | 11.3 |
|  | GBW 07603 Bush Leaves | 1.25 ± 0.15 | 1.24 ± 0.04 | 99 | 1.60 | 0.86 |
| Ni | CRM 12-2-04 Wheat bread flour | 0.3 | 0.31 ± 0.04 | 102 | 6.28 | 9.00 |
|  | GBW 07603 Bush Leaves | 1.7 ± 0.3 | 1.4± 0.1 | 80 | 3.14 | 0.74 |
|  | CRM12-2-03 Lucerne | 2.54 ± 0.18 | 2.90 ± 0.20 | 114 | 3.66 | 3.42 |
|  | GBW 10052 Green Tea | 5.4 ± 0.4 | 5.20 ± 0.05 | 96 | 0.51 | 1.11 |
|  | BCR 679 White Cabbage | 27 ± 1 | 26 ± 1 | 95 | 2.40 | 0.16 |
| Pb | CRM 12-2-04 Wheat bread flour | 0.0410 ± 0.0078 | 0.0387 ± 0.0008 | 94 | 0.97 | 7.71 |
|  | NCS ZC 73015 Milk Powder | 0.070 ± 0.020 | 0.073 ± 0.002 | 104 | 1.12 | 2.88 |
|  | GBW 10052 Green Tea | 1.60 ± 0.20 | 1.52 ± 0.06 | 95 | 1.84 | 5.11 |
|  | CRM12-2-03 Lucerne | 1.84 ± 0.17 | 1.87 ± 0.11 | 102 | 3.05 | 3.76 |
|  | GBW 07603 Bush Leaves | 47 ± 3 | 48 ± 4 | 102 | 4.37 | 0.95 |

**Table S2.**

Calculated detection limits of the method (MLODs, µg kg^-1^) and quantification limits of the method (MLOQs, µg kg^-1^) of essential and potentially toxic elements with the use of Rh as internal standard. Values are corrected for initial sample weight and final volume and reflect the actual concentration in the undigested samples.

|  | **Essential elements** | | | | | |  | | **Toxic elements** | | | |
| --- | --- | --- | --- | --- | --- | --- | --- | --- | --- | --- | --- | --- |
|  | **Ca** | **Fe** | **K** | **Mg** | **P** | **Zn** | |  | **Al** | **As** | **Ni** | **Pb** |
| **MLOD** | 499 | 0.71 | 362 | 1.7 | 228 | 1.9 | |  | 69 | 5.1 | 0.43 | 0.04 |
| **MLOQ** | 1663 | 2.37 | 1207 | 5.5 | 760 | 6.2 | |  | 230 | 17.1 | 1.43 | 0.13 |

**Table S3.**

Results of the Akaike Information Criterion (AIC) test used to identify the best-fitting probability distributions for concentration values of each element measured in botanical extracts.

| **Element** | **Best-fitting distribution** | **Best AIC** | **Second-best distribution** | **Second AIC** | Δ **AIC** |
| --- | --- | --- | --- | --- | --- |
| Ca | Lognormal | 406 | Weibull | 411 | 4.94 |
| Fe | Exponential | 252 | Weibull | 253 | 0.53 |
| K | Lognormal | 526 | Weibull | 527 | 0.66 |
| Mg | Lognormal | 389 | Weibull | 390 | 1.10 |
| P | Lognormal | 417 | Weibull | 420 | 3.37 |
| Zn | Exponential | 162 | Weibull | 164 | 1.89 |
| Al | Exponential | 219 | Lognormal | 220 | 0.79 |
| Ni | Lognormal | 412 | Weibull | 427 | 14.6 |
| As | Lognormal | 350 | Weibull | 368 | 18.0 |
| Pb | Lognormal | 290 | Weibull | 293 | 3.14 |

Note: the four candidate distributions evaluated were: lognormal, exponential, Weibull, and Pareto. The best-fitting distribution was selected based on the lowest AIC value, and model preference was assessed using the ΔAIC (i.e., the difference between the AIC of the second-best and the best model). Interpretation: ΔAIC < 3 indicates weak evidence, 3–10 moderate evidence, and >10 strong evidence in favor of the best-fitting model.^40^

**Figure S1.**


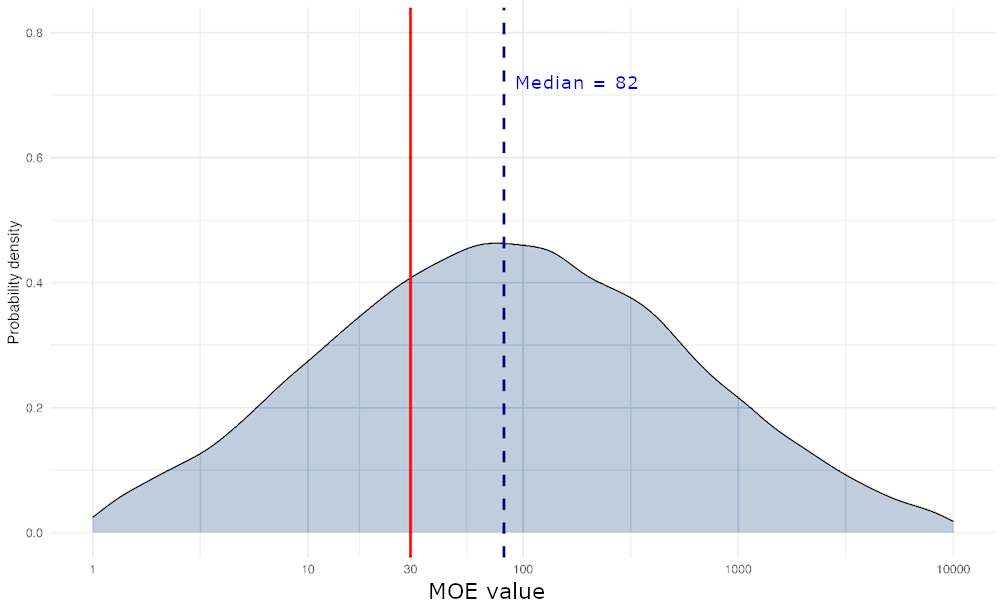
Probability distribution of Margin of Exposure (MOE, logarithmic scale) values to nickel based on acute dietary consumption of botanical extracts associated with systemic contact dermatitis in nickel-sensitized individuals
